# Adaptive Ridge Point Refinement for Seeds Detection in X-Ray Coronary Angiogram

**DOI:** 10.1155/2015/502573

**Published:** 2015-05-18

**Authors:** Ruoxiu Xiao, Jian Yang, Danni Ai, Jingfan Fan, Yue Liu, Guangzhi Wang, Yongtian Wang

**Affiliations:** ^1^Key Laboratory of Photoelectronic Imaging Technology and System, Ministry of Education of China, School of Optics and Electronics, Beijing Institute of Technology, Beijing 100081, China; ^2^Department of Biomedical Engineering, School of Medicine, Tsinghua University, Room C249, Beijing 100084, China

## Abstract

Seed point is prerequired condition for tracking based method for extracting centerline or vascular structures from the angiogram. In this paper, a novel seed point detection method for coronary artery segmentation is proposed. Vessels on the image are first enhanced according to the distribution of Hessian eigenvalue in multiscale space; consequently, centerlines of tubular vessels are also enhanced. Ridge point is extracted as candidate seed point, which is then refined according to its mathematical definition. The theoretical feasibility of this method is also proven. Finally, all the detected ridge points are checked using a self-adaptive threshold to improve the robustness of results. Clinical angiograms are used to evaluate the performance of the proposed algorithm, and the results show that the proposed algorithm can detect a large set of true seed points located on most branches of vessels. Compared with traditional seed point detection algorithms, the proposed method can detect a larger number of seed points with higher precision. Considering that the proposed method can achieve accurate seed detection without any human interaction, it can be utilized for several clinical applications, such as vessel segmentation, centerline extraction, and topological identification.

## 1. Introduction

Currently, vascular diseases are major threats to human health. Although a variety of imaging technologies exist, such as computed tomography angiography (CTA), magnetic resonance angiography (MRA), and ultrasound (US), X-ray angiography remains the gold standard for the interventional treatment of coronary artery diseases because of its high resolution and imaging speed. Foreshortening and overlapping are the major obstacles for the accurate identification of vascular structures because X-ray angiography is an integrated projection of the whole body in 3D space to 2D images. Vascular extraction technology aims to calculate the centerline, diameter, and direction vector of the vascular structure from X-ray angiograms; hence, it can provide the necessary reference for computer-aided diagnosis and treatment of vascular diseases.

To date, the widely used vascular extraction method in clinical practices is still the manual delineation method, which is very time-consuming and subjective. As its important clinical value, automatic vascular tree extraction method has been studied intensively in the past two decades, such are morphology based methods [1, 2], multiscale based methods [3, 4], edge detection based methods, and image registration based methods [5–7]. Among all methods, the tracking based methods propose to estimate centerline and diameter within the vascular boundaries, which do not need to scan the whole angiogram. Hence, the tracking based methods are usually with higher calculation efficiency than the other methods.

Generally, the tracking procedure proceeds from one or several manually delineated seed points. As the seed points are randomly selected from the angiogram, the reproducibility of the tracking algorithms are very much depended on the personal experience. Many researchers hence focus on improving the robustness of the tracking algorithm through seed optimization. Collorec and Coatrieux [8] detected seed points by scanning local extreme points and obtained a large set of seed points inside the vessels. However, extracted seed points need to be refined because of the presence of noise. While Fritzsche et al. [9] combined the global threshold optimization for improving the robustness of the seed extraction, the global threshold may also lead to a large amount of false seed points in the background. Moreover, Boroujeni et al. [10] proposed an automatic seed point detection method by detecting edge points and checking the symmetric features in its neighboring regions. After all the boundaries are detected, the center line seed points can be calculated at the symmetric center of the edge points. All the above methods have greatly promoted the automatic seed detection methods.

In this paper, a novel adaptive ridge point refinement method is proposed for seed detection in coronary angiograms. First, based on the tubular feature distribution of Hessian matrix of the angiogram, vascular structures are enhanced according to the eigenvalue distribution in multiscale space. Second, the continuity property of eigenvalue and eigenvector of a Hessian matrix in multiscale space is theoretically analyzed. Third, based on theoretical analysis, the proposed theorem of ridge point existence is utilized to design the ridge discriminant function. And the candidate ridge points are extracted according to the predefined discriminant function. Afterwards, the detected points are refined according to a self-adaptive threshold that is calculated based on the order statistics of the detected ridge points.

## 2. Method

### 2.1. Characteristic of Ideal Vascular Topology

Let *I*(*M*) represent the intensity of a point *M* in the image; then, the intensity distribution of the local feature around *M* can be calculated as follows [11]:

(1) IM+δM=IM+MT∇IM +MTHMM+oM,

where ∇*I*(*M*) = ((∂/∂*x*)*I*(*M*), (∂/∂*y*)*I*(*M*)) is the gradient of the image *I* at the point *M* with respect to the *x*-axis and *y*-axis, while (∂/∂*x*)*I*(*M*) and (∂/∂*y*)*I*(*M*) are the first-order partial derivatives of *I*(*M*) in the directions of *x* and *y*, respectively. And the Hessian matrix *H*(*M*) of point *M* can be calculated as follows:

(2) $$\begin{array}{c} H\left(M\right)=\left(\begin{array}{cc} \frac{\partial^{2}}{\partial x^{2}}I\left(M\right) & \frac{\partial^{2}}{\partial x\partial y}I\left(M\right)\\ \frac{\partial^{2}}{\partial y\partial x}I\left(M\right) & \frac{\partial^{2}}{\partial y^{2}}I\left(M\right) \end{array}\right), \end{array}$$

where (∂^2^/∂*x* ^2^)*I*(*M*) and (∂^2^/∂*y* ^2^)*I*(*M*) denote the second partial differential of *I*(*M*) in the direction of *x* and *y*, respectively, while (∂^2^/∂*x*∂*y*)*I*(*M*) and (∂^2^/∂*y*∂*x*)*I*(*M*) denote the second partial differential of *I*(*M*). If the second-order differential of *I*(*M*) is continuous, then we have (∂^2^/∂*x*∂*y*)*I*(*M*) = (∂^2^/∂*y*∂*x*)*I*(*M*). Through singular value decomposition (SVD) decomposition, two eigenvalues *λ*_1_ and *λ*_2_ (suppose *λ*_1_ ≤ *λ*_2_) and their corresponding eigenvectors *v*_1_ and *v*_2_ can be obtained from the Hessian matrix of each pixel of the angiogram. To be convenient for the followed analysis, in this study, *λ*_1_ and *λ*_2_ are named as the first eigenvalue and second eigenvalue of *H*(*M*), and *v*_1_ and *v*_2_ are denoted by first eigenvector and second eigenvector of *H*(*M*), separately.

Ideally, due to its tubular structure, the penetrating path of X-ray in the blood vessel decreases from the central axis to edge position. Therefore, the gray level distribution of the vessel in angiogram turns from dark to bright for its centerline to the edge [12]. If we look at the grey scale distribution of the blood vessel, the centerlines are rested on the ridge lines constituted by a series of ridge points. Generally, the ridge point is the local extreme with the direction vector perpendicular to the vascular direction on angiogram. Therefore, for the coronary artery in angiogram, we have the following equation [13]:

(3) $$\begin{array}{c} 0\approx \left|\lambda_{1}\right|\ll \lambda_{2}. \end{array}$$

**Figure 1:**
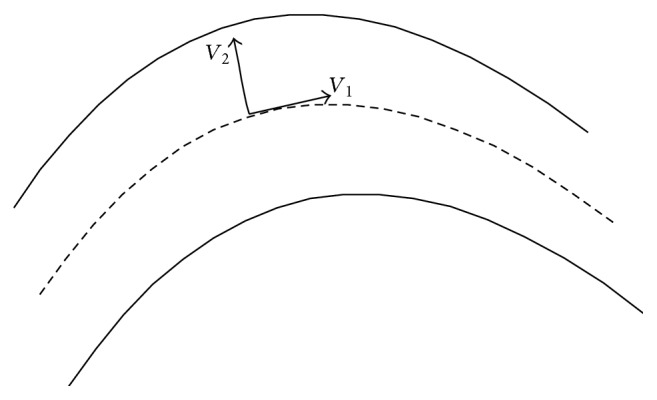
Relationship between the two eigenvectors of the Hessian matrix with respect to vascular topology.

According to the definition of the ridge, the two eigenvectors of Hessian matrix can be denoted by the tangent direction (*V*_1_) and the vertical direction (*V*_2_), as can be seen in Figure 1.

### 2.2. Multiscale Vascular Enhancement

Since the disturbance of eigenvalue or eigenvector of the Hessian matrix is in the same order [14], if the disturbance of the Hessian matrix is *ε*, then the disturbances of the corresponding eigenvalue and eigenvector are *O*(*ε*) because of the existence of noise in the angiogram. However, when zero-order disturbance is Δ*δ*, the second-order disturbance is amplified twice. In such condition, the use of eigenvalue or eigenvector will introduce a large amount of error for vessel enhancement. Hence, several researchers introduced multiscale operators, such as Gaussian scale transformation, to minimize error disturbance [3, 15, 16]. The Gaussian function has been proven to be the only kernel function in the linear scale space by Lindeberg [16, 17] and Florack et al. [18, 19]. According to the property of the Gaussian function, the multiscale space is not only linear but also satisfies several other properties, such as spatial shift invariance, noncreation of local extreme, rotational symmetry, and semigroup structure [20, 21].

In this paper, the Frangi filter [13] is utilized to enhanced vascular structure in the angiogram. For this filter, a single scale function can be defined as follows:

(4) $$\begin{array}{c} E\left(M,\sigma \right)=\left\{\begin{array}{cc} 0\;\;\;\text{if}\;\lambda_{2}<0 & \\ \exp\left(-\frac{\lambda_{1}^{2}}{2\alpha^{2}\lambda_{2}^{2}}\right)\left[1-\exp\left(-\frac{\lambda_{1}^{2}+\lambda_{2}^{2}}{2\beta^{2}}\right)\right] & \\ \;\;\;\;\text{otherwise}, & \end{array}\right. \end{array}$$

where *λ*_1_ and *λ*_2_ are the first and the second eigenvalues, while *α* and *β* are controlling coefficient. To obtain the best vascular enhancement effect, the multiscale function should have the highest response of all the sampled scales, which can be formulized as follows:

(5) $$\begin{array}{c} E\left(M\right)=\underset{\sigma_{\min}\leq \sigma \leq \sigma_{\max}}{\max}E\left(M,\sigma \right), \end{array}$$

where [*σ*_min⁡_, *σ*_max⁡_] is the predefined scaling range. Typically, *σ*_min⁡_ and *σ*_max⁡_ correspond to minimum and maximum size of vessels on image.

The image enhanced by multiscale eigenvalue of Hessian matrix is usually referred to as a vesselness image. In a vesselness image, the background of the nonvascular region is suppressed, and the vessels appear brighter than that of the original image. Moreover, the pixels along the directions of vascular centerlines are strongly enhanced and appear brighter than the ones perpendicular to the vascular direction vector. Therefore, the ridge points on a vesselness image can be extracted to stand for the seed points from the extraction of blood vessels.

### 2.3. Continuity Analysis of Vesselness Image

In this study, the candidate seed points are extracted by detecting ridge points in a vesselness image based on the differential continuity of the image, which includes gradient and Hessian matrix together with its corresponding eigenvalue and eigenvector for each pixel. Usually, they are not located at integer coordinates and should be computed by interpolation. However, they should be continuous when being interpolated. Therefore, the continuity of the abovementioned differential information is very important for seed detection in the angiogram.

In traditional methods of differential information analysis, the differential information of the image is assumed to be continuous. Theoretically, an image can be described as a 2D continuous signal obtained from the optical sensor by light integration. As such, the zero-order gray scale information is a continuous function for the coordinates. To further utilize the differential information, this study theoretically analyzed the continuous property of gradient, eigenvalue, and eigenvector.

Suppose that *f*(*x*, *y*; *σ*) represents an image convolved by a Gaussian function with kernel of *σ*; then we have the following equation:

(6) $$\begin{array}{c} \frac{\partial }{\partial x}f\left(x,y;\sigma \right)⊗g\left(x,y,\sigma \right)=f\left(x,y;\sigma \right)⊗\frac{\partial }{\partial x}g\left(x,y,\sigma \right), \end{array}$$

since

(7) $$\begin{array}{c} \frac{\partial }{\partial x}g\left(x,y,\sigma \right)=-\frac{x}{\sigma^{2}}g\left(x,y,\sigma \right). \end{array}$$

Then, the continuity of an image in Gaussian space will be transformed into the continuity of the image after Gaussian convolution. Therefore, we first discuss the continuity of the image after Gaussian smoothing in the following section.

According to Lemma A.1 (as can be seen in the section of appendix), we found that any 1D continuous function will be infinitely differentiable when it is convoluted with the Gaussian function. Similarly, the vesselness image, which is a two-dimensional continuous function, will be infinitely differentiable with the application of Gaussian smoothing. Essentially, if the vesselness image is at least differentiable in the second-order, its gradient vector and Hessian matrix are also continuous. As can be inferred from Lemma A.3, the two eigenvalues of Hessian matrix of vesselness image are single eigenvalue and the corresponding eigenvectors of these two eigenvalues are continuous according to Lemma A.2. Therefore, all the related differential terms, including gradient and Hessian matrix together with its corresponding eigenvalue and eigenvector of vesselness image, are continuous.

### 2.4. Seed Point Detection

In traditional methods, the detection of local maxima points in the image is a common method of seeding. However, the points on the centerline are essentially not the local maxima points in any direction. Instead, they are the local maxima points on the directions perpendicular to the centerline. In this paper, the ridge points are extracted as candidates of seed points. The definition of ridge point could be derived from the definition of local extreme point, which can be described as follows [13].

**Definition 1 .**

Let *f* : *ℝ* ^*n*^ → *ℝ* represent a second-order continuous function. A point *x* ∈ *ℝ* ^*n*^ is a local extreme point for *f* if (*v* · ∇)*f*(*x*) = 0 for every direction *v*; that is, ∇*f*(*x*) = 0. The extreme point can be classified as follows: (1) *x* is a local minimum point, if (*v* · ∇)^2^ *f*(*x*) > 0 for every direction *v*; (2) *x* is a local maximum point, if (*v* · ∇)^2^ *f*(*x*) < 0 for every direction *v*. The corresponding function value *f*(*x*) at the extreme point *x* is named as the extreme value.

According to the Hessian matrix, the above definition of local extreme point can be described as follows.

**Definition 2 .**

Let *f* : *ℝ* ^*n*^ → *ℝ* be a second-order continuous function. A point *x* ∈ *ℝ* ^*n*^ is as follows: (1) a local minimum point for *f* if ∇*f*(*x*) = 0 and the Hessian matrix of *x* is positive definite (all the eigenvalues are positive); (2) a local maximum point if ∇*f*(*x*) = 0 and the Hessian matrix of *x* is negative definite (all the eigenvalues are negative).

A *n*-*d* type ridge point is the local maximum point in *n*-*d* orthogonal directions in *n*-dimensional space of which the definition is as follows.

**Definition 3 .**

Let *f* : *ℝ* ^*n*^ → *ℝ* be a second-order continuous function. A point *x* ∈ *ℝ* ^*n*^ is a *n*-*d* type ridge point if and only if [*v*_1_,…,*v*_*d*_]^*T*^∇*I*(*x*) = 0 and *λ*_*d*_ < 0, where, *λ*_1_,…, *λ*_*n*_  (*λ*_1_ ≤ ⋯≤*λ*_*n*_) are the eigenvalues of hessian matrix of point *x* and their corresponding eigenvectors are denoted by *v*_1_,…, *v*_*n*_, and 1 ≤ *d* ≤ *n*.

To extract ridge points on the image, we need to find all the points to meet the conditions that [*v*_1_,…,*v*_*d*_]^*T*^∇*I*(*x*) = 0 and *λ*_*d*_ < 0. For angiograms, images are two-dimensional data, ridge points are 1-type, and we need to detect the points that satisfy (*v*_1_)^*T*^∇*I*(*x*) = 0 and *λ*_1_ < 0. According to the density of the real number, detecting all the ridge points within a limited time is impossible because ridge points are usually located on subpixel coordinates rather than on integer coordinates. In this paper, we obtain a sufficient number of ridge points based on the analysis of the gradient vector and the Hessian matrix at discrete pixels.

*Ridge Point Existence Criterion*. Assume that *I*(*x*) is a vesselness image, for a point (*x*, *y*) and its neighbor points (*x* + 1, *y*), (*x*, *y* + 1), and (*x* + 1, *y* + 1). There must be a ridge point  (*ξ*, *η*)  (*x* ≤ *ξ* ≤ *x* + 1, *y* ≤ *η* ≤ *y* + 1) between them if the conditions are satisfied as follows:

(8) max⁡⁡v1∇Ix,y,v1∇Ix+1,y,  v1∇Ix,y+1,v1∇Ix+1,y+1>0,min⁡⁡v1∇Ix,y,v1∇Ix+1,y,  v1∇Ix,y+1,v1∇Ix+1,y+1<0,max⁡⁡λ1x,y,λ1x+1,y,  λ1x,y+1,λ1x+1,y+1<0,

where ∇*I*(*x*, *y*) is the gradient vector at (*x*, *y*) and *λ*_1_(*x*, *y*) and *v*_1_(*x*, *y*) are the first eigenvalue and the first eigenvector of Hessian matrix at (*x*, *y*).

**Proof**

According to the Lemmas A.2 and A.3 and Theorem A.4, we know that since *I*(*x*) is two-order continuous, ∇*I*(*x*, *y*) and *H*(*x*, *y*) are continuous. *λ*_1_(*x*, *y*) is a single eigenvalue; therefore, *λ*_1_(*x*, *y*) and *v*_1_(*x*, *y*) are continuous for (*x*, *y*).

Thus

(9) max⁡⁡v1∇Ix,y,v1∇Ix+1,y,  v1∇Ix,y+1,v1∇Ix+1,y+1>0,min⁡⁡v1∇Ix,y,v1∇Ix+1,y,  v1∇Ix,y+1,v1∇Ix+1,y+1<0.

According to the intermediate value theorem of continuous function, there is a point (*ξ*, *η*)  (*x* ≤ *ξ* ≤ *x* + 1, *y* ≤ *η* ≤ *y* + 1), which meets *v*_1_∇(*ξ*, *η*) = 0.

And since *λ*_1_(*ξ*, *η*) can be achieved by linear interpolation of *λ*_1_(*x*, *y*), *λ*_1_(*x* + 1, *y*), *λ*_1_(*x*, *y* + 1), and *λ*_1_(*x* + 1, *y* + 1),

(10) λ1ξ,η=ω3ω1λ1x,y+1−ω1λ1x+1,y +1−ω3 ·ω2λ1x,y+1+1−ω2λ1x+1,y+1,ω2λ1x,y+1+1−ω2λ10≤ω1,ω2,ω3≤1.

We have

(11) max⁡⁡λ1x,y,λ1x+1,y,   λ1x,y+1,λ1x+1,y+1<0.

Then *λ*_1_(*ξ*, *η*) < 0.

According to Definition 3, the point (*ξ*, *η*) is a ridge point. And according to the ridge point existence criterion, the ridge points can be detected by scanning the vesselness image line by line. In this paper, we take the pixel point (*x*, *y*) as the ridge point (*ξ*, *η*) to be a seed point. It can not only save the interpolation burden but also guarantee the extraction accuracy of the seed point.

### 2.5. Seed Point Refinement

A large amount of seed points located in vascular boundaries can be detected using the proposed method. However, a number of candidate seed points located on the background, which are denoted as pseudo seed points, can be observed because of the influence of noise. In this study, an automatic seed point refinement method is proposed based on the area gray scale distribution of the detected seed points. With *P* as the set of the sample points located in the area of the detected candidate seed points, a self-adaptive threshold function can be defined as follows:

(12) $$\begin{array}{c} T=m\left(P\right)-\omega \cdot s\left(P\right), \end{array}$$

where *m*(*P*) is the median intensity value of arranged *P* and *s*(*P*) is the median of the absolute value of all the points of *P* minus *m*(*P*). *ω* is a weight factor, which controls the noise and sensitivity of the intensity.

**Figure 2:**
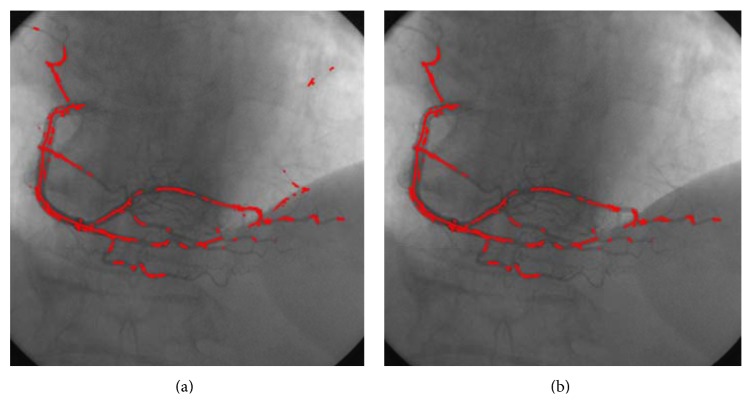
Seed point calculation results in an angiogram. (a) Results of the ridge based method. (b) Results after seed refinement.

Figure 2(a) shows the extracted candidate seed points on an angiogram based on the proposed ridge detection method. As shown in the figure, a large number of seed points are detected inside the vascular structures. However, some of them are still detected in the background.  Figure 2(b) shows the refined results of the proposed method. As observed, the pseudo seed points located in the nonvascular region are removed, and most of the calculated seed points are inside vascular boundaries, whereas some of them are located near the vascular centerlines.

## 3. Experimental Results

To validate the performance of the proposed method, a series of coronary angiograms acquired from a Philips Digital Imaging device at Beijing Chaoyang Rea-Cross Hospital were used. All angiograms have 512 × 512 resolution. The experiments were carried out on a desktop computer with an i7-2600 processor and 16 G memory, and the proposed method is compared with the other two traditional seed point detection algorithms.

**Figure 3:**
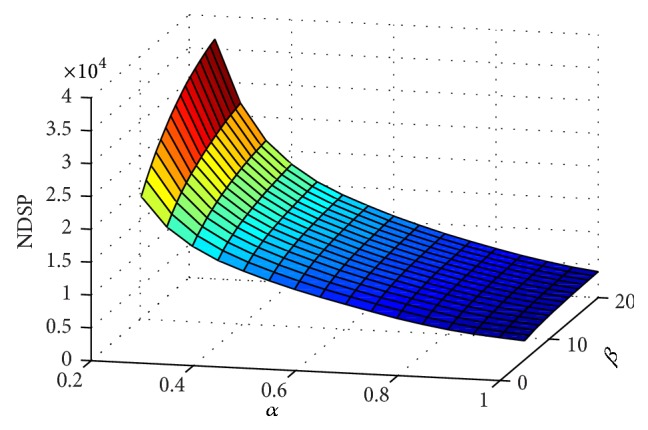
Relationship between NDSP and enhancement parameters *α* and *β*.

Figure 3 was designed to evaluate the number of detected seed point (NDSP) with respect to the multiscale enhancement filtering parameters of *α* and *β*. For the experiment, the sampling ranges of *α* and *β* are set at [0.25,1] and [5,20], respectively, where the sampling step of *α* and *β* is set at 0.05 and 5. In this study, NDSP is defined as the number of detected seeds with respect to the enhancement response value larger than a predefined threshold of *τ*. In this experiment, the value of *τ* is set at 30. From the figure, if *β* is set constant, the NDSP values decrease quickly with increasing *α*. If *α* is set constant, the NDSP values increase slowly with increasing *β*. When *β* is comparatively small, a large amount of small enhanced noise appears in the angiogram. Hence, suppressing the noise while preserving the effect of enhancement during seed extraction is important.

**Figure 4:**
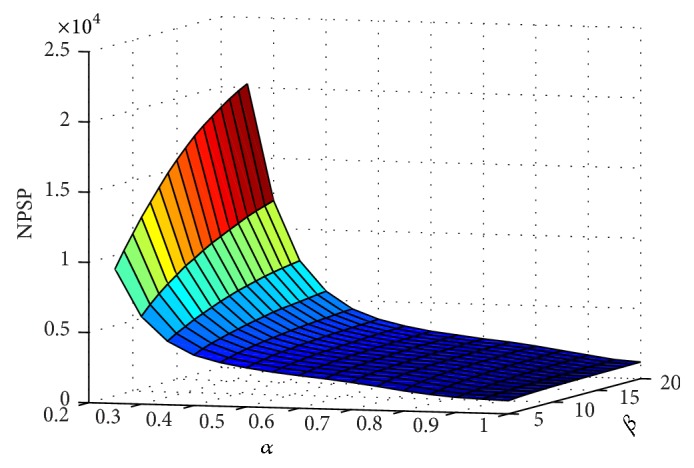
Relationship between NPSP and the enhancement parameters of *α* and *β*.

To obtain the optimal enhancement parameters, the structures of coronary arteries in the angiogram were manually delineated from the background. As such, the number of pseudo seed points (NPSP) that rest in the background can be effectively quantified.  Figure 4 demonstrates the relationship between NPSP and the enhancement parameters of *α* and *β*. From the figure, if *β* is set constant, NPSP first deceases rapidly and then slowly with increasing *α*. On the other hand, if *α* is set constant, NPSP increases slowly with increasing *β*.

To obtain the optimal enhancement parameters, the false detection ratio (FDR) is defined based on NDSP and NPSP as follows:

(13) $$\begin{array}{c} \text{FDR}=\frac{\text{NDSP}}{\text{NPSP}}. \end{array}$$

**Figure 5:**
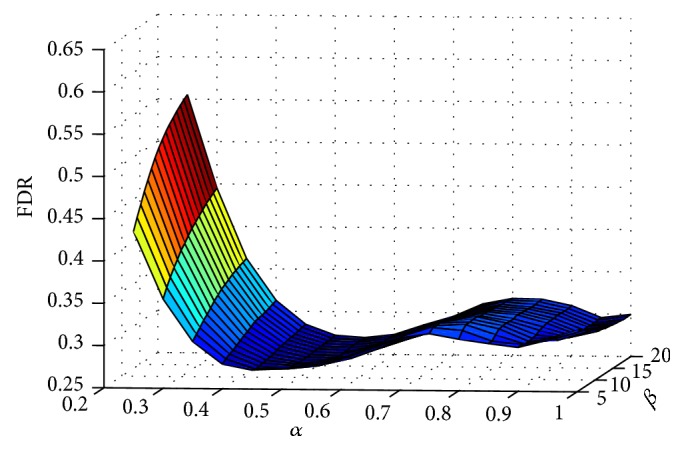
Relationship between the FDR and the enhancement parameters of *α* and *β*.

Figure 5 demonstrates the relationship between FDR and the enhancement parameters of *α* and *β*. From the figure, if *β* is set constant, FDR first deceases rapidly and then increases slowly with the increasing *α*. On the other hand, if *α* is set constant, NPSP first deceases and then increases slowly with increasing *β*. Therefore, there is a local minimum value of FDR corresponding to the optimal enhancement parameters of *α* and *β*.

**Figure 6:**
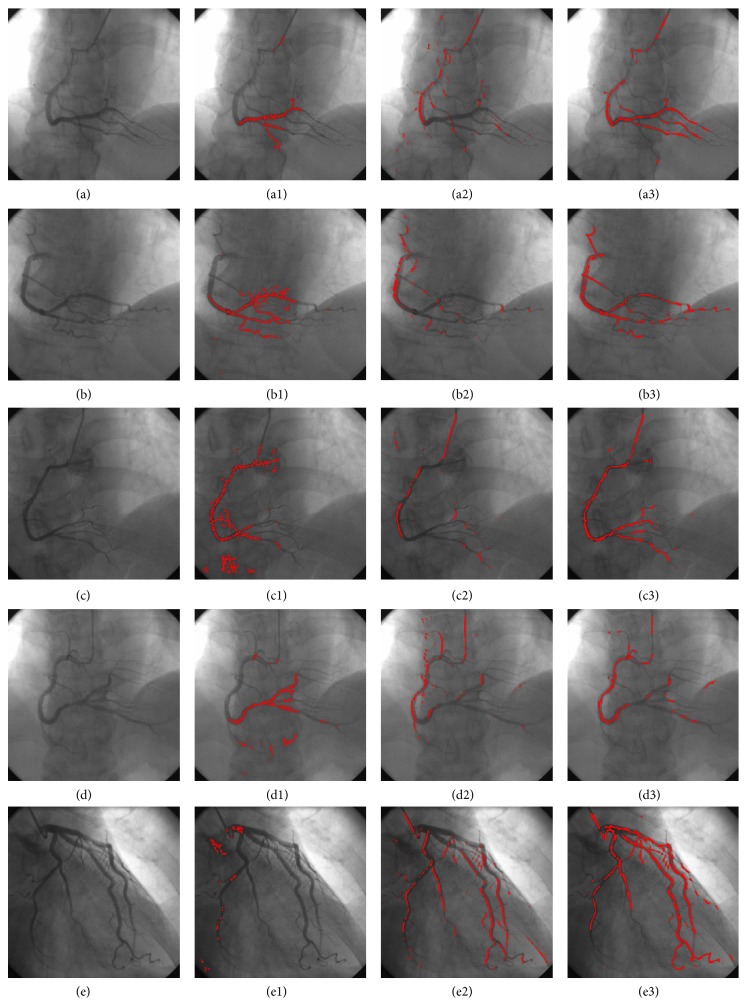
Seed point extraction results of five groups of data sets. The first to the fourth columns correspond to the source angiograms, segmentation results of Fritzsche, Boroujeni, and the proposed methods. The first to the fifth rows correspond to five different data sets.

To quantify the performance of the proposed method, the proposed seed point detection algorithm is compared with the methods proposed by Fritzsche et al. [9] and by Boroujeni et al. [10].  Figure 6 shows the seed extraction results from all the methods. The first to fourth columns correspond to the original angiograms, the results of Fritzsche, Boroujeni, and those of proposed method, respectively. The first to fifth rows correspond to five different data sets. For the Fritzsche method, only a few seed points are detected, which are mostly distributed in local parts of the vessels. Moreover, some detected points rest in the background. For the Boroujeni method, a number of detected seed points in the first four images are very small. Although more seed points are detected in the last image, detecting seed points in some of the major branches is difficult. Evidently, our proposed method can detect more seed points inside vascular boundaries than the other two methods. Furthermore, the detected seed points are evenly distributed in the whole vasculature. Hence, it can be used more appropriately for the tracking procedures for centerline extraction.

To evaluate the proposed automatic seed point detection algorithm, three general measures of precision, recall, and *f*-measure are used in this paper. And the seed detection precision can be quantified by the percentage of correct seeds accounting for all the generated seeds; then we have the following equation:

(14) $$\begin{array}{c} P=\frac{\text{TP}}{\text{TP}+\text{FP}}, \end{array}$$

where TP denotes the true positives (it is the total number of the detected seed points that are located inside the true vessels), while FP is the number of false positives (it is the number of the detected seed points that are located in the background).

The result of recall denotes the percentage of corrected seed points that can be detected by the proposed algorithm, and we have

(15) $$\begin{array}{c} R=\frac{\text{TP}}{\text{TP}+\text{FN}}, \end{array}$$

where FN denotes the number of false negatives; it is the total number of true seed points that are wrongly discarded by the refinement calculation procedure.

To balance between the precision and recall, the *f*-measure is proposed as follows:

(16) $$\begin{array}{c} \text{FM}=\frac{2\times R\times P}{R+P}. \end{array}$$

Also, the total number of seed points and the number of vascular branches that can be detected are utilized to evaluate the preformation of algorithms, and they are denoted by *N*1 and *N*2, respectively. *N*1 shows the ability that how much seed points can be detected by the algorithm. The greater of *N*1 means more vascular point will be detected in the image; while the great of *N*2 means more vascular branches will be detected in the image.

Table 1 compares the seed point detection results of Fritzsche, Boroujeni, and the proposed method over five groups of data sets. The mean values of *N*1 of the Fritzsche, Boroujeni, and the proposed methods are 1127, 1845, and 2072, respectively. Obviously, the proposed method obtained more seed points than the other two methods. In the same manner, the mean values of precision of the Fritzsche, Boroujeni, and the proposed methods are 88.1%, 96.3%, and 98.2%, respectively. It indicates that only a few detected points of the proposed method are outside of the vascular boundaries, while the mean values of recall and *f*-measure of the Fritzsche, Boroujeni, and the proposed methods are almost the same, but the proposed algorithm is slightly higher than the other two methods. The mean values of *N*2 of the Fritzsche, Boroujeni, and the proposed methods are 5, 7, and 8, respectively. Clearly, the proposed method detects a larger number of vascular branches than the other two methods. It can be concluded that the Boroujeni method is better than the Fritzsche method, while the proposed method outperforms the other two methods with respect to accuracy and the ability of branch detection.

## 4. Conclusion

This study proposes a novel automatic seed point detection method for X-ray angiographic images, which can be further utilized for vascular segmentation as well as centerline extraction. In study, the continuous properties of the eigenvalue and eigenvector are analyzed in depth. Based on the ridge point existence theorem, a novel discriminative function is proposed for candidate seed point detection from the multiscale Gaussian response of the angiographic image. The candidate seeds are refined according to the intensity distribution of neighboring pixels in the scanning lines. Furthermore, this study also discussed the optimal parameters for accurate seed detection. The study introduces five discrimination standards to quantify seed detection ability and evaluate the performance of different seed detection methods. The experiments demonstrate that the proposed method is very effective and robust for seed point detection in angiographic images with mean values of 98.2% and 2072 for the precision and number of detected seed points, respectively. Considering that the proposed method is fully automatic and with high detection ability, it can be utilized for fast centerline extraction as well as structure measurement for coronary arteries in clinical practice.

## Figures and Tables

**Table 1:** Comparison of the seed point detection results of the Fritzsche method, Boroujeni method, and the proposed method over five groups of data sets.

Data	Fritzsche method					Boroujeni method					Proposed method				
	*P*	*R*	FM	*N*1	*N*2	*P*	*R*	FM	*N*1	*N*2	*P*	*R*	FM	*N*1	*N*2
Data1	0.985	0.999	0.992	720	5	0.959	0.989	0.974	1272	9	0.994	0.999	0.996	1561	10
Data2	0.901	0.992	0.944	1488	7	0.973	0.998	0.985	1310	6	0.988	0.999	0.993	1760	9
Data3	0.912	0.981	0.945	1881	5	0.973	0.997	0.985	1131	5	0.990	0.999	0.994	1655	6
Data4	0.871	0.985	0.924	1129	4	0.936	0.989	0.962	1648	5	0.955	0.995	0.974	1079	6
Data5	0.736	0.948	0.829	416	2	0.976	0.992	0.984	3862	8	0.981	0.996	0.988	4306	11
Mean	0.881	0.981	0.927	1127	5	0.963	0.993	0.978	1845	7	0.982	0.998	0.989	2072	8

## Appendix

Lemma A.1 A.1. For any *f*(*x*) ∈ *C*, then *F*(*x*) = *f*(*x*)∗*g*(*x*) ∈ *C* ^*∞*^, where $g\left(x\right)=\left(1/\sqrt{2\pi }\right)e^{-x^{2}/2}$.

Proof Consider

(A.1) Fx=fx∗gx=∫−∞+∞ftgx−tdt=12π∫−∞+∞fte−x−t2/2dt=12πe−x2/2∫−∞+∞ftdt    +∫−∞+∞ftextdt+∫−∞+∞fte−t2/2dt∶=12πF1+F2+F3,

where *F*_1_ and *F*_2_ are infinitely differentiable functions parameterized by *x* and *F*_3_ is a constant. Therefore *F*(*x*) = *f*(*x*)∗*g*(*x*) ∈ *C* ^*∞*^.

Lemma A.2 A.2. Let *A*, Δ*A* ∈ *C* ^*n*×*n*^, where *A* = {*a*_*ij*_} and Δ*A* = {Δ*a*_*ij*_}. If *λ* is a single eigenvalue of *A* and *V* is the corresponding eigenvector of *λ*, then for eigenvalue *λ*(*A* + Δ*A*) and its corresponding eigenvector *V*(*A* + Δ*A*) of *A* + Δ*A*, it satisfied that *λ*(*A* + Δ*A*) → *λ* and *V*(*A* + Δ*A*) → *V*, when Δ*A* → 0.

Proof Since the eigenvalue of the Hessian matrix is a continuous function for all the matrix elements and *λ* is a single eigenvalue of *A*, then for the eigenvalue *λ*(*A* + Δ*A*) of *A* + Δ*A*, *λ*(*A* + Δ*A*) → *λ* when Δ*A* → 0. And for a sufficiently small ‖Δ*A*‖, *λ*(*A* + Δ*A*) is a single eigenvalue of *A* + Δ*A*. Since *λ* is a single eigenvalue of *A*, according to the theorem of Jordan canonical form, we have rank(*λE* − *A*) = rank(*A*) − 1, where *E* is an *n* × *n* identity matrix. Hence, we can find certain *i* and *j*, for which the *n* − 1 order nonsingular matrix *A*_*n*−1_^*^ can be obtained by removing the elements of the *i*th row and the *j*th column of *A*. According to

(A.2) $$\begin{array}{c} AV=\lambda V, \end{array}$$

where *V* = (*v*_1_,…,*v*_*n*_)^*T*^, the coefficient matrix of the system

(A.3) −∑m=1m≠jm=nakm−δkmλvm=akj−δkjλvj, k≠i,

is nonsingular, where

(A.4) $$\begin{array}{c} \delta_{ij}=\left\{\begin{array}{cc} 1, & i=j\\ 0, & i\neq j. \end{array}\right. \end{array}$$

Without loss of generality, we assume that *v*_*j*_ = 1. For a sufficiently small ‖Δ*A*‖, *λ*(*A* + Δ*A*) is a single eigenvalue of *A* + Δ*A*, and rank(*λE* − (*A* + Δ*A*)) = rank(*A* + Δ*A*) − 1. The *n* − 1 order matrix (*A* + Δ*A*)_*n*−1_^*^, which is obtained by removing the elements of the *i*th row and the *j*th column of *A* + Δ*A*, is also nonsingular. Hence, the system of linear equations

(A.5) −∑m=1m≠jm=nakm+Δakm−δkmλA+ΔAvmA+ΔA =akj+Δakm−δkjλA+ΔAvjA+ΔA, k≠i,

have a unique solution *v*_1_(*A* + Δ*A*),…, *v*_*j*−1_(*A* + Δ*A*), *v*_*j*+1_(*A* + Δ*A*),…, *v*_*n*_(*A* + Δ*A*), and they are continuous functions of Δ*A*. If we choose *v*_*j*_(*A* + Δ*A*) = *v*_*j*_ = 1, there will be

(A.6) VA+ΔA=v1A+ΔA,…,vj−1A+ΔA,  vjA+ΔA,vj+1A+ΔA,…,  vj−1A+ΔA,vnA+ΔAT⟶V,

when Δ*A* → 0.

Lemma A.3 A.3. If the image *I*(*x*, *y*) is second-order continuous, then the two eigenvalues *λ*_1_ and *λ*_2_ of Hessian matrix of the point (*x*, *y*) are unequal, and their corresponding eigenvalues *v*_1_ and *v*_2_ are orthogonal to each other.

Proof If *I*(*x*, *y*) ∈ *C* ^2^, then *I*_*xy*_ = *I*_*yx*_; that is, the hessian matrix *H*(*x*, *y*) of (*x*, *y*) is symmetrical. According to the properties of real symmetric matrices, the two eigenvectors of *H*(*x*, *y*) are orthogonal. The two eigenvalues can be obtained by solving the characteristic polynomial $\left|\begin{array}{cc} \lambda -I_{xx} & -I_{xy}\\ -I_{yx} & \lambda -I_{yy} \end{array}\right|=0$ as follows:

(A.7) $$\begin{array}{c} \lambda^{2}-\left(I_{xx}+I_{yy}\right)\lambda +I_{xx}I_{yy}-I_{xy}^{2}=0. \end{array}$$

The two eigenvalues are equal if and only if

(A.8) $$\begin{array}{c} {\left(I_{xx}+I_{yy}\right)}^{2}-4\left(I_{xx}I_{yy}-I_{xy}^{2}\right)=0. \end{array}$$

From (A.8) we have

(A.9) $$\begin{array}{c} {\left(I_{xx}-I_{yy}\right)}^{2}+4I_{xy}^{2}=0. \end{array}$$

*H*(*x*, *y*) has two equal eigenvalues if and only if

(A.10) Ixx=Iyy,Ixy=Iyx=0.

By solving (A.10), we have

(A.11) Ix=c1,Iy=c2,

where *c*_1_ and *c*_2_ are constant. To satisfy (A.11), *I*(*x*, *y*) ≡ *c*, where *c* is constant; that is, the intensity of image *I*(*x*, *y*) is constant, which is in conflict with our application. Therefore, for each point (*x*, *y*) on image *I*(*x*, *y*), the two eigenvalues *λ*_1_ and *λ*_2_ of the Hessian matrix *H*(*x*, *y*) are unequal.

Theorem A.4 A.4. If an image *I*(*x*, *y*) ∈ *ℂ* ^2^, for any point (*x*, *y*) on *I*(*x*, *y*), its eigenvalues and eigenvectors of Hessian matrix are continuous.

Proof If *I*(*x*, *y*) ∈ *ℂ* ^2^, the Hessian matrix *H*(*x*, *y*) is $\left(\begin{array}{cc} I_{xx} & I_{xy}\\ I_{yx} & I_{yy} \end{array}\right)$, where the elements *I*_*xx*_, *I*_*xy*_, *I*_*yx*_, and *I*_*yy*_ are all continuous. According to the definition of eigenvalues, the two eigenvalues *λ*_1_(*x*, *y*) and *λ*_2_(*x*, *y*) are two real roots of the characteristic polynomial of *H*:

(A.12) $$\begin{array}{c} f\left(\lambda \right)=\left|\begin{array}{cc} \lambda -I_{xx} & -I_{xy}\\ -I_{yx} & \lambda -I_{yy} \end{array}\right|=0. \end{array}$$

For continuous functions, their roots are also continuous; that is *λ*_1_(*x*, *y*) and *λ*_2_(*x*, *y*) are continuous. Therefore, the eigenvalues of Hessian matrix of image are continuous.
